# Visual anticipation biases conscious decision making but not bottom-up visual processing

**DOI:** 10.3389/fpsyg.2014.01443

**Published:** 2015-01-30

**Authors:** Zenon Mathews, Ryszard Cetnarski, Paul F. M. J. Verschure

**Affiliations:** ^1^Synthetic, Perceptive, Emotive and Cognitive Systems Group, Department of Technology, Information and Communication, Center of Autonomous Systems and Neurorobotics, Universitat Pompeu FabraBarcelona, Spain; ^2^Institucio Catalana de Recerca i Estudis Avançats, Passeig Llus CompanysBarcelona, Spain

**Keywords:** decision making, displacement (psychology), saccades, dynamical systems, information processing, visual perception, predictive modeling

## Abstract

Prediction plays a key role in control of attention but it is not clear which aspects of prediction are most prominent in conscious experience. An evolving view on the brain is that it can be seen as a prediction machine that optimizes its ability to predict states of the world and the self through the top-down propagation of predictions and the bottom-up presentation of prediction errors. There are competing views though on whether prediction or prediction errors dominate the formation of conscious experience. Yet, the dynamic effects of prediction on perception, decision making and consciousness have been difficult to assess and to model. We propose a novel mathematical framework and a psychophysical paradigm that allows us to assess both the hierarchical structuring of perceptual consciousness, its content and the impact of predictions and/or errors on conscious experience, attention and decision-making. Using a displacement detection task combined with reverse correlation, we reveal signatures of the usage of prediction at three different levels of perceptual processing: bottom-up fast saccades, top-down driven slow saccades and consciousnes decisions. Our results suggest that the brain employs multiple parallel mechanism at different levels of perceptual processing in order to shape effective sensory consciousness within a predicted perceptual scene. We further observe that bottom-up sensory and top-down predictive processes can be dissociated through cognitive load. We propose a probabilistic data association model from dynamical systems theory to model the predictive multi-scale bias in perceptual processing that we observe and its role in the formation of conscious experience. We propose that these results support the hypothesis that consciousness provides a time-delayed description of a task that is used to prospectively optimize real time control structures, rather than being engaged in the real-time control of behavior itself.

## 1. Introduction

Theories of consciousness can be grouped with respect to their stance on embodiment, sensori-motor interaction, prediction and information integration (Verschure, 2013). In this context, especially the notion of prediction seems to play a key role in linking the conscious experience of an intentional agent to its physical environment and embodiment (Hesslow, 2002; Merker, 2005; Revonsuo, 2006; Barsalou, 2008). Examination of the relation between top-down predictive and bottom-up perceptual processes can produce new insights into the functional significance of conscious experience (Hohwy, 2012). In our case we address this question from the perspective of the Distributed Adaptive Control theory of mind and brain which proposes that conscious experience provides a serialized task description that is used to optimize parallel control loops that drive real-time performance (Verschure, 2013). We have investigated how bottom up and top down streams of information processing dynamically couple and or uncouple (i.e., dissociate) depending on the demand for cognitive resources dedicated to the consciously pursued task. We hypothesize that under increasing cognitive load there will be an increasing dissociation between perceptual processes and conscious experience. We assess whether the functional significance of this dissociation can be interpreted within the predictive brain framework, which suggests that conscious experience represents the top-down predictions of sensory states rather than reflecting the actual bottom-up states of sensory systems (Hohwy, 2012; Verschure, 2012). In this view it is through simulation that an internal world, or self-generated virtual reality (Revonsuo, 1995), can appear in consciousness, freeing the organism from its immediate physical environment and its specific contingencies (Edelman, 2001; Verschure, 2012). However, the functional significance of conscious experience cannot be understood without investigating the origins of the representations that eventually populate this internal world. Within the predictive brain framework two alternative hypotheses can be considered, either the content of conscious experience represents “generative models” (i.e., top-down predictions) or the internal world is populated by prediction errors (i.e., information not included in the generative models but rather expressed in their discrepancy with sensory states derived from the world). To discriminate between the two hypotheses, we designed a psychophysical task based on prior theoretical work (Mathews et al., 2011), where subjects were asked to detect stimulus displacements of otherwise predictably behaving visual stimuli. Subjects' responses were measured on three levels of information processing: conscious displacement detections and fast and slow saccadic detections. By increasing the cognitive load, without affecting the difficulty of the displacement detection task, we were able to assess whether the three types of responses were equally affected when processing events which violated subjects' predictions. This allowed us to examine how the prediction error, i.e., conflict between sensory information and top down prediction, differentially affected these different levels of information processing. By analyzing the predictive properties of non-detected (i.e., dissociated) events we were able to quantify the extent to which conscious experience depends on sensory information or on a predictive model.

### 1.1. Predictive brain framework

The idea that the information processing of the brain is organized around prediction has reached prominence in the so-called Bayesian Brain and predictive coding frameworks (Verschure, 2003; Friston, 2005; Barsalou, 2008; Clark, 2013). In this view, core structures of the brain including the thalamo-cortical and the cortico-basal ganglia system and the cerebellum are engaged in forms of hierarchical Bayesian inference, extracting generative models of both sensory inputs and the consequences of action, across multiple time scales and modalities (Hesslow, 2002; Cisek and Kalaska, 2010; Lau and Rosenthal, 2011). In neurophysiological terms, top-down connections are suggested to convey the content of these generative (predictive) models, while bottom-up signals convey prediction errors between actual and predicted sensory states (Rao and Ballard, 1999; Bar, 2007; Friston, 2010; Mathews et al., 2011). How inference is exactly affecting perception, cognition and consciousness is currently being debated. For instance, the exact relation between predictive processing and consciousness remains poorly understood although some correlations between the two have been reported in coma patients where an impairment of top-down functional connectivity was observed (Boly et al., 2011). Moreover, there is no consensus on which sorts of predictive models give rise to conscious contents, and which do not. For instance, the cerebellum which comprises about 70% of the neurons of the central nervous system, generates adaptive predictions of discrete events (Kawato, 1999), while it is commonly assumed that this structure operates outside of the window of conscious experience (but see Laurens et al., 2013 for an interesting exception). Furthermore, it is unclear what the exact relations are between probabilistic representations as postulated by the Bayesian brain and the unitary and singular nature of experience (Merker, 2005). In addition, the impact of predictions on the conscious states of a system conceived as a hierarchical Bayesian inference machine is debated. On one hand one could argue that subjective states should be dominated by the content of top-down predictions (Hohwy et al., 2008; Clark, 2013) while others have argued that it is exactly the bottom-up prediction errors that should define the conscious scene facilitating error correction (Blakemore et al., 1999; Pally, 2005; Mudrik et al., 2011). Moreover, the Bayesian brain perspective does not have a clear funtional role for conscious experience. Hence, it is of some relevance to experimentally assess whether these two perspectives on the role of inference in the structuring of both unconscious processing and conscious experience are contradictory or complementary (Nir and Tononi, 2010). Several experimental paradigms (see Bar, 2009 for a review) and a few mathematical frameworks (Grossberg, 2009) have been proposed to measure and model the top-down effects of prediction at different levels of visual processing. Nevertheless, an experimental paradigm and theoretical framework to directly identify the dynamic influence of prediction at different levels of visual perception has yet to be proposed. Moreover, beyond some accounts of anticipatory modulation of physiological responses in frontal brain areas (Summerfield et al., 2006), it still remains unclear how prediction/anticipation directly affects perception at a neurophysiological level. Here we present a combined theoretical and experimental approach that allows achieving this objective in the context of a perceptual task.

The idea that perception is defined through predictive models of environmental causes of sensory input enjoys a rich pedigree (Tolman, 1932; Craik, 1943; Helmholtz, 1860; Gregory, 1961; Barlow, 1972). Experimental signatures of explicit or implicit anticipation of stimuli are manifested in diverse perceptual tasks, including the reduced response delays observed in visual processing due to subliminal priming (Kiesel et al., 2007) and changes in reaction time and precision of the smooth pursuit of objects and motion (Barnes et al., 2000; Winges and Soechting, 2011). At the level of the neuronal substrate, this notion has found support in physiological accounts of the influence of long-range cortical projections in modulating the activity of primary sensory areas (Cox et al., 2004; Ekstrom et al., 2008), the anticipatory modulation of bottom-up visual responses (Summerfield et al., 2006) and in the integration of bottom-up processes including at the single neuron level (Fries et al., 2001; Ekstrom et al., 2008). Also models of perception widely use the principle of the minimization of the reconstruction error between sensory input and its prediction (Grossberg, 1980; Verschure et al., 1992; Montague et al., 1995; Dean and Porrill, 2008; Duff and Verschure, 2010). Given this experimental and theoretical support, the question is whether this inference system that mediates between sensation and conscious experience follows a monolithic hierarchical structure or rather dynamically configures itself dependent on task constraints.

### 1.2. Anticipatory fields for data association

At an abstract level the notion of hierarchical inference seems to capture the functional aspects of mind, brain and behavior. However, when translating it to the level of physical implementation and real-time, real-world interaction a number of important questions emerge. For instance, in considering the role of prediction in perception it is usually seen as a monolithic process involving a single prediction and error generation mechanism (Spratling, 2010). However, computational work has shown that physiologically realistic hierarchical models of visual systems can be generated by systems that optimize specific objectives defined through a top-down error signal (Wyss et al., 2006). In addition, the computational principles of systems underlying perceptual learning might differ from those driving behavioral learning (Verschure, 2012). Where the former can be seen to follow the statistics of inputs, the latter has to process the significance of transient and intermittent reinforcement signals in the face of varying goals and environmental conditions (Verschure et al., 2014). This illustrates that it cannot be excluded that predictions and their underlying representations are generated differently and independently at various levels of perceptual an cognitive processing, from the mechanisms driving rapid saccades to processes underlying conscious decisions. For instance, subjects consciously performing a cognitive task, displayed a perceptual learning dependent bias in the processing of subliminally presented visual stimuli that was associated with enhanced responses in numerous neuronal structures including the hippocampus and the neocortex (Turk-Browne et al., 2010). Building on prior theoretical work (Mathews et al., 2011), we first ask whether signatures of predictions of sensory events, measured as “anticipatory fields,” can be observed in visual decision making tasks and investigate if such biases affect bottom-up and top-down perceptual processes. To this end, we designed a psychophysical experimental paradigm to investigate the hierarchical organization of the processes underlying a basic perception action loop. Using a psychophysical method called “reverse correlation” (Ahumada, 1996), we define spatial locations around stimulus movement trajectories were stimulus displacements are not detected by the subjects. These spatial locations are referred to as “anticipatory fields” and are calculated independently for three levels of information processing: conscious detections of displacements and fast and slow sacadic detections. Our model proposes that observations that fall outside of these regions (“anticipatory fields”) are exceptions and call for the allocation of resources, i.e., attention or action, in order to correct for prediction errors (See Materials and Methods). Effectively, this means that the concepts that structure perception are defined in a feed-forward fashion through the association of individual observations, i.e., data association. In turn these concepts regulate data acquisition in a top-down fashion by specifying regions in data space where future relevant events are expected. In order to assess this process structure and the relationship between the different stages of processing, we manipulated the cognitive load of the subjects using an additional cognitive task. Following the Distributed Adaptive Control theory of mind and brain (Verschure et al., 1992) we distinguish three distinct levels of processing that are increasingly more dependent on memory and information integration: fast and slow saccades and conscious decisions. Our results show that cognitive load has direct, quantifiable and dynamic influences on the parallel visual anticipations generated by the subjects as measured in their fast and slow saccades and conscious decisions. We subsequently model the observed phenomena using Bayesian dynamical systems theory to capture both the parallel and the top-down bias of anticipation in human visual perception. Our results provide concrete evidence for influences of prediction in perception at higher levels of information processing and for dynamic uncoupling of unconscious and conscious processes depending on task demands and behavioral goals.

## 2. Materials and methods

### 2.1. Experimental procedure

**Figure 1:**
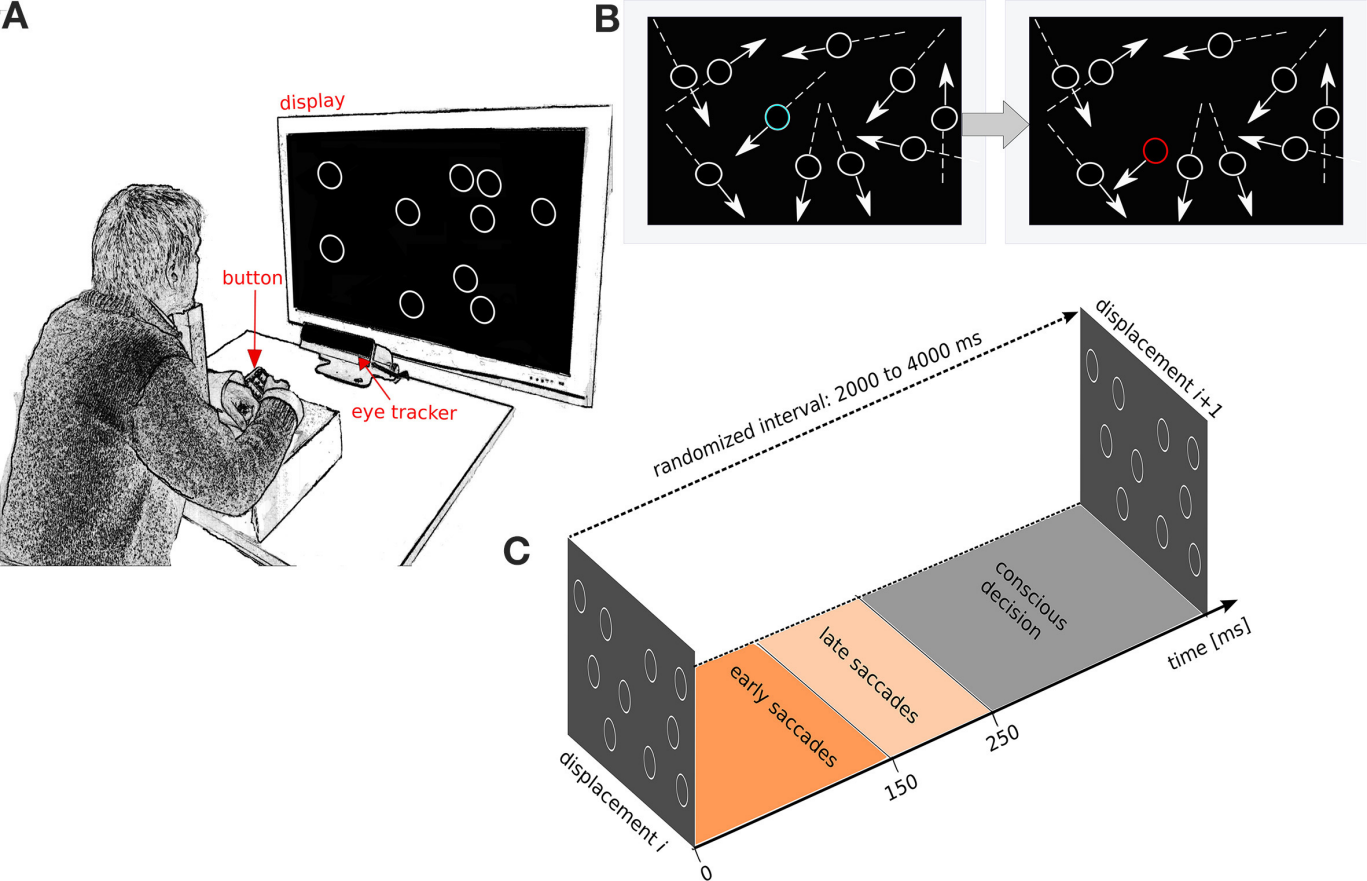
**Experimental setup and experimental protocol. (A)** Subjects face a screen with the head stabilized on a chin rest. The display shows linearly moving circular items that deflect off the sides of the display. Subjects report detected displacements with a button press. **(B)** Illustration of a displacement event. Left: the constellation of the moving circles before the displacement. Right: constellation after displacement. Movement direction is indicated by arrows. The displaced item is shown in blue before the displacement and in red after the displacement. The colors, arrows and lines are only for illustration purposes, and not used in the experiment. **(C)** Schematic of the time windows used to define fast saccades, slow saccades and conscious decisions after displacement. The inter-displacement times are randomized between 3000 and 8000 ms.

13 subjects observed a fixed number of identical non-filled white circles (*n* = 10, referred to as items) on a gray background (Figure 1A). Each item followed a linear path at a constant speed and bounced against the display boundaries. The movement speed of each circular item was 14°C/s and held constant. Once every T seconds, one of the moving items was displaced (i.e., jumped) from its linear trajectory and then continued its linear motion at the same speed and direction prior to displacement (Figure 1). Inter-displacement time T was randomized between 3000 and 8000 ms to avoid automated rhythmic responses. The displacement was randomized in two ways: first, the item to be displaced was randomly chosen from all items excluding those closer than 3° to a boundary. Second, the displacement angle was drawn from a uniform distribution from 0 to 360° and the displacement distance was randomly chosen between 2.5 and 7.5°. After the displacement the item continued movement on the same linear path. The primary task of the subjects was to press a button whenever they perceived a displacement of an item (Figure 1). Linear movements of items were used in order to control the movement predictions generated by the subjects (Lisberger et al., 1987). By using several simultaneously moving circular items, we avoided visual habituation during smooth-pursuit of a single linear movement (Eggert et al., 2009). Subjects were not instructed to fixate or to saccade, but were merely instructed to look freely anywhere on the screen. The task was to press the button if a displacement was seen. The subjects were not instructed to respond quickly and no feedback on performance was given. The subjects were 13 university students (5 female) 27 ± 5.11 SD age. All had normal or corrected-to-normal vision. Subjects provided written consent before the experiments. A session consisted of 9 trials, 3 min each (3 trials for each of 3 cognitive load conditions defined below). The experimental design allowed to modulate the cognitive load without affecting the perceptual load (Camos and Barrouillet, 2004). In the low cognitive load condition the subject solely performed the above displacement detection task (referred to hereafter as the low load task). For the medium cognitive load condition the subjects were instructed to continuously recite aloud the alphabet in their mother tongue while performing the above psychophysics task (the medium load task). For the high cognitive load condition the subjects were instructed to continuously recite aloud the alphabet in their mother tongue in reverse order skipping every other letter (high load task). Recalling less automatized chains (alphabets in reverse order and skipping every other letter) is known to induce higher cognitive loads than more automatized chains (like the alphabet in forward order) (Camos and Barrouillet, 2004). The order of low, medium and high cognitive load tasks were randomized for each subject. After each trial subjects could rest for 1 min and take their head off the chin rest.

### 2.2. Experimental setup

We used the Tobii X120 eye tracker (Tobii Technology, 2014) that tracks gaze position at 120 Hz. The visual stimulus presentation environment was developed using the Unity 3D engine and ran at 250 Hz, with a 60 Hz refresh rate of the screen. The button press and the circular item movements were logged and time synchronized with the eye tracking data. The LCD screen measured 115^*^65 cms with 1920 × 1080 pixels resolution. The subject sat head stabilized at 110 cm from the screen midpoint, giving a 2.4° radius of the circular items used as visual stimuli. The contrast ratio between the white moving item and the gray background was 2.27 in RGB scale. The screen was covering 63.07° horizontally and 32.92° vertically of the subject field of view. The movement speed of the circular items was 14°. Eye tracker calibration was performed once for each subject before the experiments. Calibration error was below 2°. Subjects were alone in the controlled experimental space and were instructed to recite aloud the alphabets so that it was audible to the experimenter in the nearby separated space.

### 2.3. Data analysis

The gaze data collected from the eyetracker was classified into two distinct types of events: fixations and saccades. These events were distinguished based on point-to-point velocities of gaze: low velocity points were marked as a fixation while high velocity ones were marked as a saccade. In order to reduce noise in the velocities and thus make the classification method more accurate we applied a clustering algorithm over the raw gaze data based on Tobii studio solution (Tobii Technology, 2014). The algorithm moved each point to a centroid calculated from a mean position of points found within a 96 ms time window and below a fixed distance threshold. This procedure created well defined, spatially and temporally, clusters of fixations separated by saccade gaze points. We studied two kinds of saccades and computed the anticipatory fields for each of them separately. First we looked at the fast saccades that occur below 250 ms after displacement of a stimulus. Second, we looked at the slow saccades that occur between 250 and 800 ms after displacement. We defined a displacement as detected by an fast/slow saccade, if the saccade toward the displacement location occurred in the above defined time windows. To distinguish between saccades toward the displacement and random locations we used a cosine measure describing the angle between a vector from gaze position to displacement position and a vector of a real saccade. All data analysis was performed using Python toolboxes (sciPy, numPy).

### 2.4. Psychophysical reverse correlation

**Figure 2:**
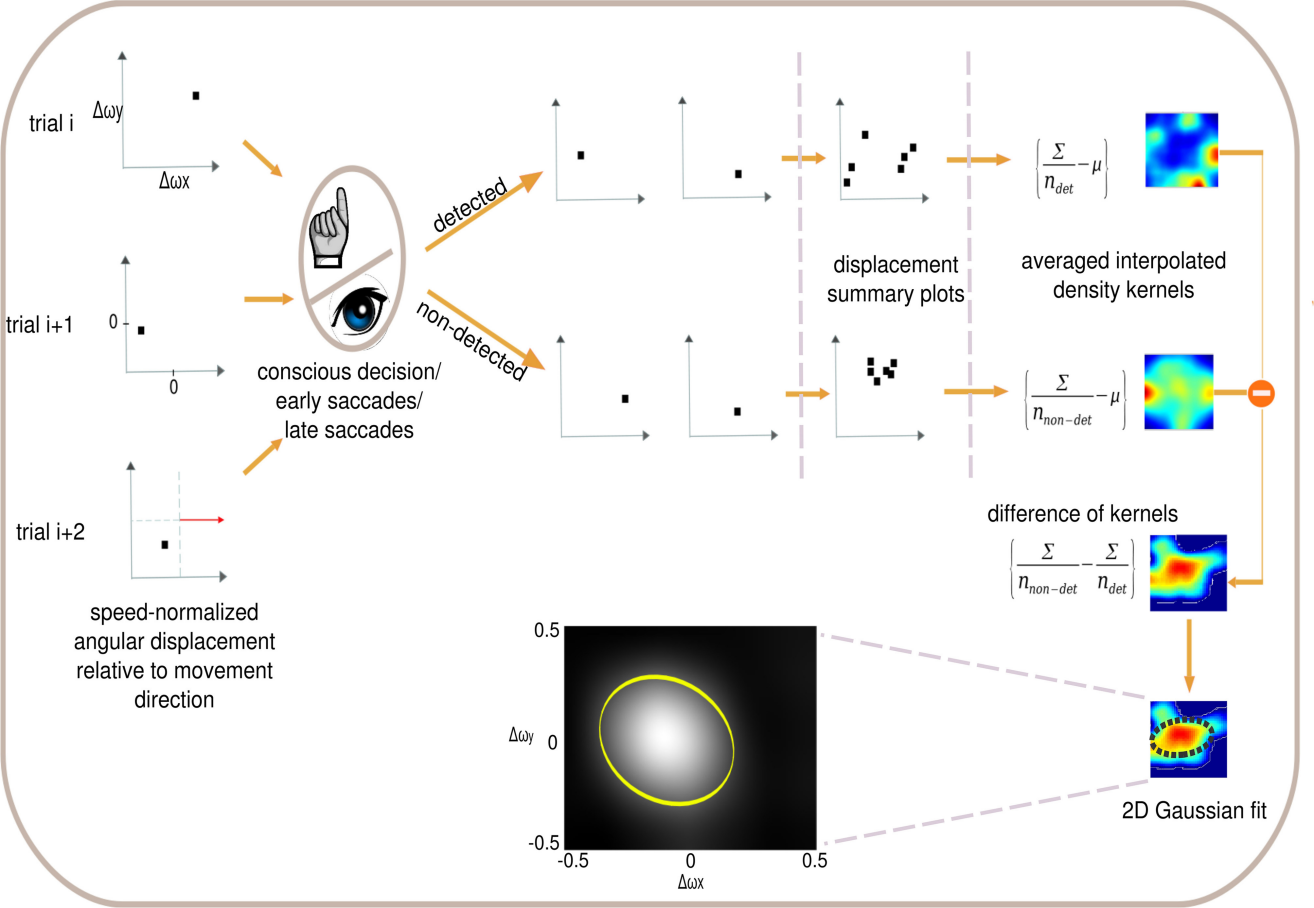
**Psychophysical reverse correlation**. Each displacement trial (left) is corrected for movement direction defining the x−axis (red arrow). X and Y axis indicate horizontal and vertical angular displacements respectively after correction for direction of stimulus movement. Each trial is sorted in terms of the three detection levels (conscious decision, fast saccade and slow saccade) and the density kernels of detected and non-detected trials are computed separately. Further, the detected trial's kernel is subtracted from the non-detected kernel and the error ellipse is computed for this difference of kernels. This error ellipse is the psychophysical kernel or anticipatory field for that specific response class. We analyze four properties of the anticipatory fields: area, shift, eccentricity and orientation. Inset shows the anticipatory field for a single subject for the fast saccades in the low cognitive load task.

Reverse correlation has been used in psychophysical studies to characterize observer strategies in visual tasks (Ahumada, 1996) and in physiological studies to characterize neural responses to visual stimuli (Victor, 2005). Reverse correlation has proven to be a powerful technique for seeking relationships between a high-dimensional variable (e.g., an image) and a categorical variable (two-choice decision or neural response) (Victor, 2005). Here we tailored psychophysical reverse correlation to analyze the conscious decisions, and fast and slow saccades of our subjects. Each event is a displacement that is plotted as a point on a direction-corrected coordinate system (Figure 2, left), where the positive x axis is the linear movement direction of the circular item. We then sort the stimuli according to the detected and non-detected displacements (using either button presses, fast or slow saccades as the detection criterion). We subsequently compute the average detection and non-detection densities and use data interpolation to yield a two-dimensional probability distribution for detection and non-detection densities separately. The difference between the two probability distributions (non-detected versus detected) is computed and fitted with a 2D Gaussian distribution. The covariance ellipse of the Gaussian distribution is referred to as the psychophysical kernel or the anticipatory field.

## 3. Model

We consider the Joint probabilistic data association (JPDA) algorithm as the model underlying human visual data association in dynamic predictable scenarios (Bar-Shalom and Fortmann, 1988). JPDA is a single-scan approximation to the optimal Bayesian filter, which associates latest observations to known targets sequentially. JPDA enumerates all possible associations between observations and targets at each time step and computes the association probabilities β_*jk*_, which is the probability that the *j*-th observation originated from the *k*-th target. Given such association probabilities, the target state is estimated by Kalman filtering (Kalman, 1960) and this conditional expectation of the state is weighted by the association probability. Let *x*^*k*^_*t*_ indicate the state of target *k* at time step *t*, ω_*jk*_ the association event where the observation *j* is associated to target *k* and *Y*_1:*t*_ stays for all the observations from time step 1 to time step *t*. Using apriori knowledge about the world (e.g., state transition matrix(*A*), process noise (*Q*), measurement matrix (*H*), control-input model (*B*) and the control input-vector ($\hat{u}$) of the Kalman filter) and the current state of the *target*, a prediction is made for each *target*. At timestep *t*, for each *target k*, we compute the state prediction, its covariance and the measurement prediction as follows

(1) $$\;{\tilde{x}}_{k}^{k}=A{\hat{x}}_{t\;-\;1}^{k}+B{\hat{u}}_{t\;-\;1}^{k}$$

(2) $${\tilde{P}}_{t}^{k}=A{\hat{P}}_{t\;-\;1}^{k}A^{T}+Q_{t\;-\;1}^{k}$$

(3) $${\tilde{y}}_{t}^{k}=H{\tilde{x}}_{t}^{k}$$

Then the state of the target can be estimated as:

(4) $$E(x_{t}^{k}|Y_{1:t})=\sum_{\omega }E\left(x_{t}^{k}|\omega ,Y_{1:t}\right)P(\omega |Y_{1:t})$$

(5) $$\;=\sum_{j}E\left(x_{t}^{k}|\omega_{jk},Y_{1:t}\right)P(\omega_{jk}|Y_{1:t})$$

where ω_*jk*_ denotes the association event where observation *j* is associated to target *k* and ω_0*k*_ denotes the event that no observation is associated to target *k*. Therefore, the event association probability is β_*jk*_ = (ω_*jk*_|*Y*_1:*t*_). JPDA computes a anticipatory gate for each target using the Kalman innovation of new observations. It only considers observations inside the anticipatory gate for each target. The ellipsoidal anticipatory gate using the Kalman filter is discussed in section “Psychophysical reverse correlation.”

We consider the linear state evolution model for state dynamics of target *x* at time *k*:

(6) $$x_{k}=Ax_{k\;-\;1}+Bu_{k\;-\;1}+\rho_{p}$$

where ρ_*p*_ is the process noise with time-invariant covariance matrix *Q*, *B* is the control-input model and *u*_*k*_ the control vector. The well-known linear Kalman filter prediction and estimation steps is used to update the state. The ellipsoidal anticipatory gate is optimal for the above linear observation model^1^ with additive noise:

(7) z=Hx+φ

where φ is the zero Gaussian measurement error with *p*(φ) = 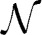
(φ; 0, *R*) and is independent of the state *x*. *H* is the observation model which maps the true state space into the observed space. The state probability density function is Gaussian *p*(*x*) = 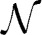
(*x*; $\hat{x}$, *P*). The validity of measurement *y*_*i*_ is determined from its innovation with the predicted observation:

(8) $$\nu =y_{i}-H\hat{x}$$

with the covariance *S* = *R* + *HPH*^*T*^. The anticipatory gate is computed by gating the Mahanalobis distance (the normalized innovation squared (NIS)):

(9) $$\nu^{T}S^{-1}\nu <M_{d}$$

*M*_*d*_ is the threshold for an innovation dimension *d* and can be computed efficiently since the NIS follows a chi-square probability density function. e.g., to compute the probability that *j*% of true associations are accepted, *M*_*d*_ is obtained from

(10) $$\frac{j}{100}=P(\frac{d}{2},\frac{M_{d}}{2})$$

where

(11) $$P(a,b)=\frac{1}{\Gamma (a)}\int_{0}^{b}\text{​}e^{-t}t^{a\;-\;1}dt$$

is the incomplete gamma function (Press et al., 1992). The anticipatory field (commonly known as *validation gate*) defines a region of acceptance such that (100 − *j*)% of true measurements are rejected given that the measurements *y*_*i*_ are distributed according to 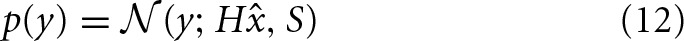


This formulation avoids the necessity to model clutter, which is usually very hard to model, and also unlikely associations are eliminated. For non-linear anticipatory gates for non-Gaussian models see Bailey et al. (2006).

## 4. Results

### 4.1. Behavioral results

**Figure 3:**
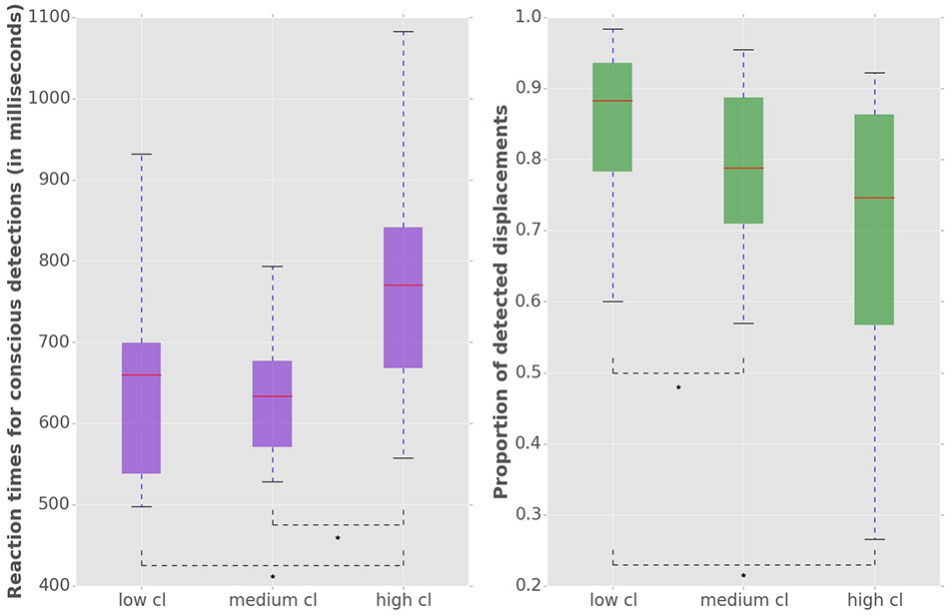
**Left:** Reaction times for consciously detected displacements. Reaction times were measured from the onset of the stimulus displacement untill the subject's response with a key press. “Cl” stands for “cognitive load.” The asterisk mark conditions where differences were statistically significant (*p* < 0.05). For the low cognitive load condition mean reaction time was 653 (±) 127 ms, for medium 654 (±) 122 ms for high 764 (±) 134 ms. **Right:** Proportion of consciously detected displacements. The proportion represents the amount of consciously detected displacements out of all displacement events. The average proportion of detected displacements (i.e., accuracy) was 0.86 (±) 0.11 for low, 0.80 (±) 0.10 for medium and 0.67 (±) 0.22 for high cognitive load.

We used a repeated measures MANOVA and a post hoc test with Bonferroni correction to identify the within subject differences in the accuracy and latency of conscious detections (Figure 3). MANOVA with a Greenhouse-Geisser correction determined that mean reaction times differed significantly between conditions [*F*_(1.455, 17.458)_ = 20.849, *p* = 0.009]. An increase of cognitive load increased the response times, by 111 ms (±) 25 SE (*p* = 0.002) from low to high load and by 110 ms (±) 15 SE (*p* < 0.001) from medium to high load. We observed a significant decrease of the conscious detection rate [*F*_(1.149, 12.641)_ = 8.918, *p* = 0.009]. The post hoc test revealed that an increase of cognitive load decreased the response accuracy, by 6.1% (±) 1.7 SE (*p* = 0.013) from low to medium cognitive load and by 19.0% (±) 5.5 SE (*p* = 0.016) from low to high cognitive load. The accuracy decreased from medium to high cognitive load by 13.0% (±) 5.5 SE however this difference was not statistically significant (*p* = 0.116). This observation provides a first indicator of a dissociation between bottom-up saccades and conscious decision processes.

### 4.2. Gaze results

**Figure 4:**
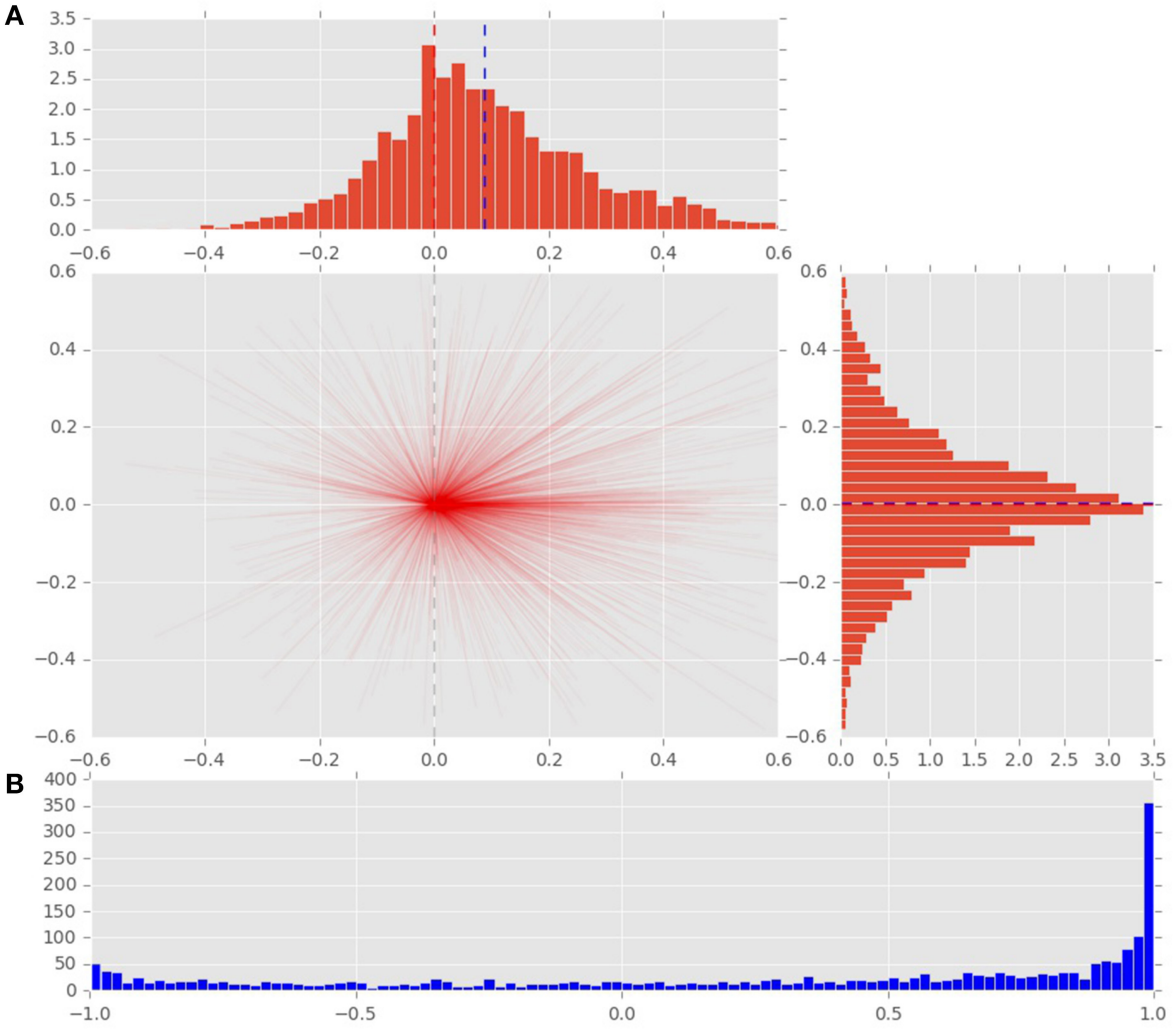
**(A)** Distribution of saccade vectors in the time window following a displacement event. The positive x-axis represents direction toward the displacement position. The magnitude of saccade vector represents the absolute size of gaze shift. Vertical blue line represents the mean of the distribution, red line marks the 0 value. **(B)** The cosine of the angle between the x-axis and the saccade vector represents the effect of the displacement on the gaze shifts. The cosines of value 1 represent saccades directed at the displacement location.

**Figure 5:**
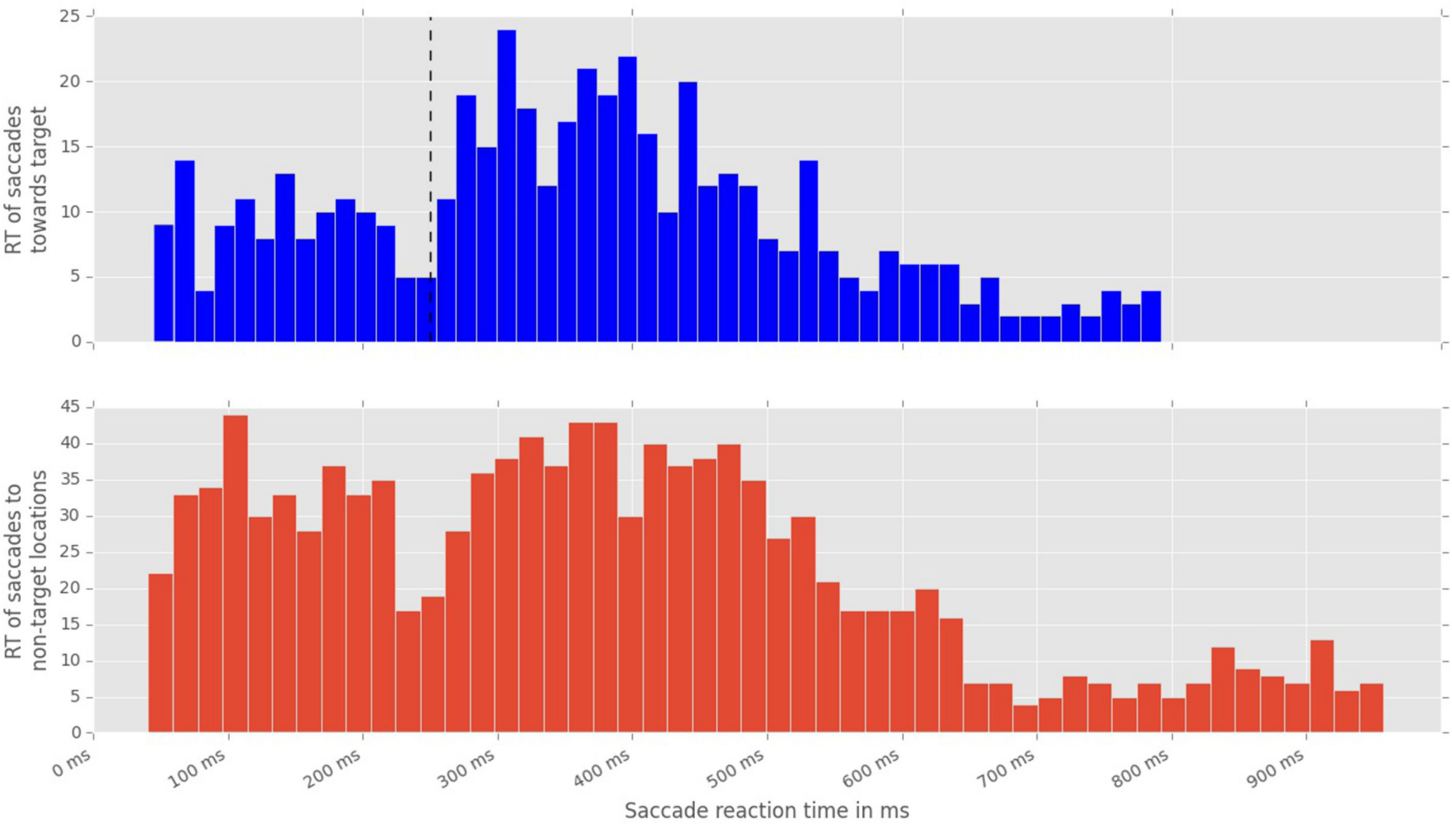
**Distribution of saccadic response times, measured from the onset of stimulus displacement untill the beggining of the first fixation following the displacement event. Top:** latencies of saccades toward a displacement event. Black line marks the division between fast and slow saccades. **Bottom:** latencies of saccades toward non-displacement locations.

**Figure 6:**
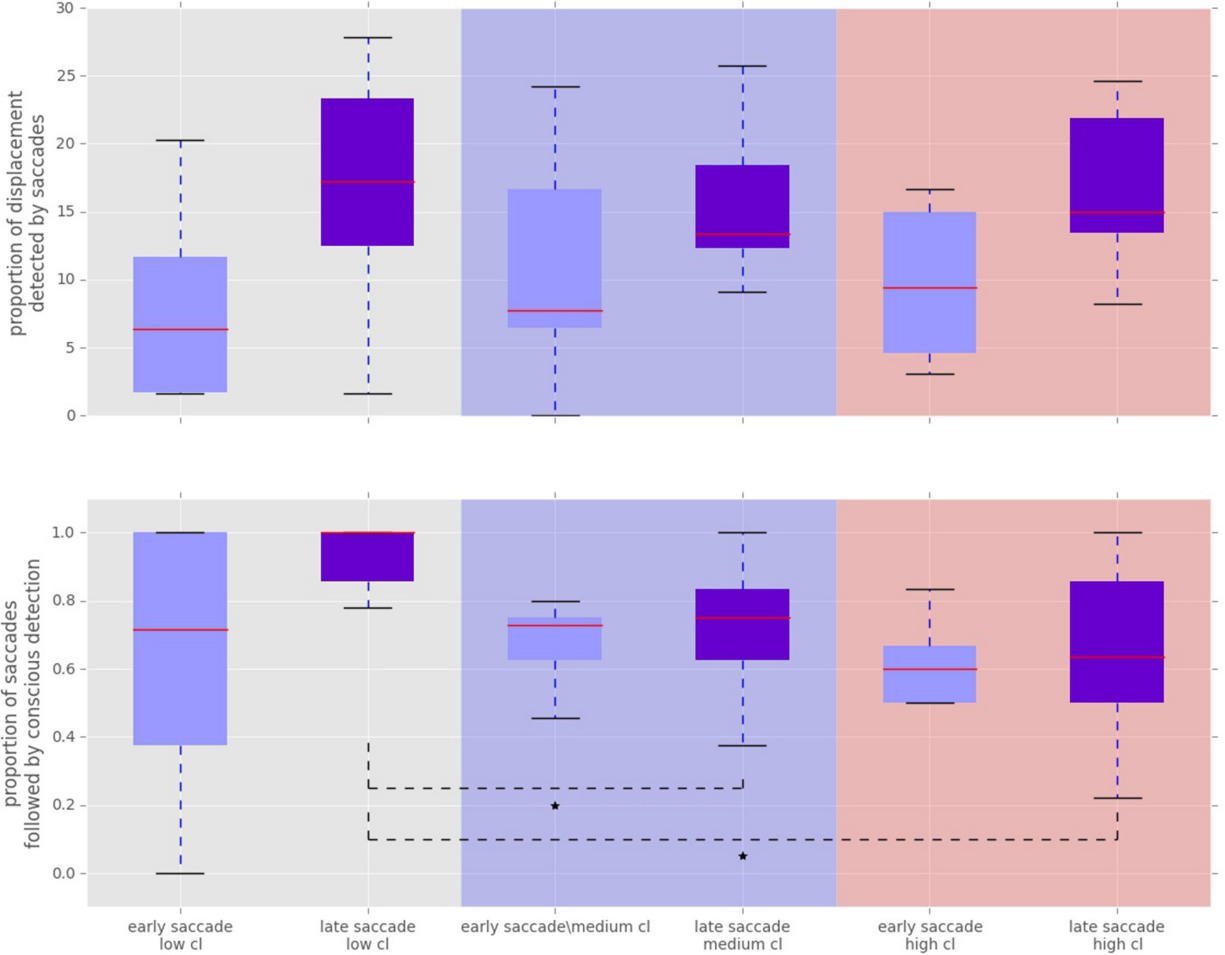
**Proportion of displacements wich were followed by a saccade towards the displacement location**. “Cl” stands for “cognitive load” condition. The asterisks mark conditions where differences were statistically significant (*p* < 0.05). **Top:** Proportion of displacements detected by fast and slow saccades. The proportion of displacements detected by fast and slow saccades did not change across conditions [χ^2^_(2)_ = 3.500, *p* = 0.174 for fast saccades and *F*_(1.81, 21.789)_ = 0.319, *p* = 0.709 for slow saccades]. **Bottom:** Proportion of saccadically detected events also followed by conscious detection. This proportion was significantly different between the conditions [χ^2^_(2)_ = 10.844, *p* = 0.004]. The median amount of late saccadic detections followed by conscious detections was 1.0 in low condition, 0.76 for the medium and 0.63 for the high condition.

To analyze the effect of the displacement event on the gaze behavior, focused on the gaze shifts in the time window following an event. We identified the first fixation cluster that ended after the event. These fixation clusters represent the last locations where subjects was looking before the event occurred. The first fixation cluster that started after the displacement represented the shift of attention temporally related to the displacement. To measure the extent to which the events attracted attention we measured parameters of gaze shift (i.e., saccades) between the last fixation before and first fixation after the displacement. The difference between the fixation position before and after the displacement event, represents a vector of a saccade in the temporal window of interest (Figure 4). We translate the origin all of these vectors to the center of a two dimensional coordinate system and rotate them around the angle between the x-axis and the displacement location (Figure 4A). Thus, the positive direction along the x-axis represents the gaze shifts toward the displacement location. The magnitude of each vector represents the absolute size of the gaze shift. We observe that the distribution of the x-components of gaze shift vectors is positively skewed towards the positive tail of the distribution. Using a K-squared test we determined that the distribution is significantly different from normal (skewness = 0.38, *p* < 0.01). The angle between the represented vector and the x-axis represents the accuracy of the gaze shift relative to the position of the displacement. By measuring the cosine between the real gaze shift vector and the optimal trajectory towards the displacement (i.e., positive x-axis) we obtain a measure which shows the effect of displacement events on gaze shifts. The cosine value of 1 represents a saccade exactly towards the event location, while –1 represents and anti-saccade. In Figure 4B we show the distribution of the cosine where the majority of the data points occur around the value of 1. Using the Kolmogorov-Smirnov test we compare this distribution to a hypothetical random uniform distribution based on values between −1 and 1 which would occur if there was no effect of displacement on the gaze shift. The test confirms that the displacements significantly affected the directions of saccades (*D* = 0.21, *p* < 0.001). We observe that the reaction time of saccades, i.e., the latency of the gaze shift following the displacement event showed a bi-modal distribution. We divided the saccadic responses based on their latency into fast saccades occurring below 250 ms and slow saccades occurring between 250 and 800 ms (Figure 5). We hypothesized that under increasing cognitive load, there will be a dissociation between bottom-up perceptual and top-down conscious processes. To elucidate this dissociation, we analyzed the relation between fast and slow saccades and conscious decisions after displacements. We observe that although the number of fast [χ^2^_(2)_ = 3.500, *p* = 0.174] and slow [*F*_(1.81, 21.789)_ = 0.319, *p* = 0.709] saccadically detected displacements does not change with cognitive load, there is a significant decrease in the proportion of slow saccades that are followed by conscious decisions [χ^2^_(2)_ = 10.844, *p* = 0.004] (Figure 6). A Wilcoxon signed-rank test using Bonferroni correction showed that there is a significant decrease in the median of saccades followed by conscious detection from the low (*M* = 1.0) to the medium (*M* = 0.76, *p* = 0.016) and from the low to the high (*M* = 0.63, *p* = 0.016) cognitive load condition. i.e., with increasing cognitive load, an increasing number of slow saccades are not followed by conscious detections. This again suggests a dissociation of bottom-up saccadic and top-down conscious decision making processes. To further understand this phenomenon we investigated the specific features of the detected and non-detected displacements in the three cognitive load conditions.

### 4.3. Psychophysical reverse correlation

**Figure 7:**
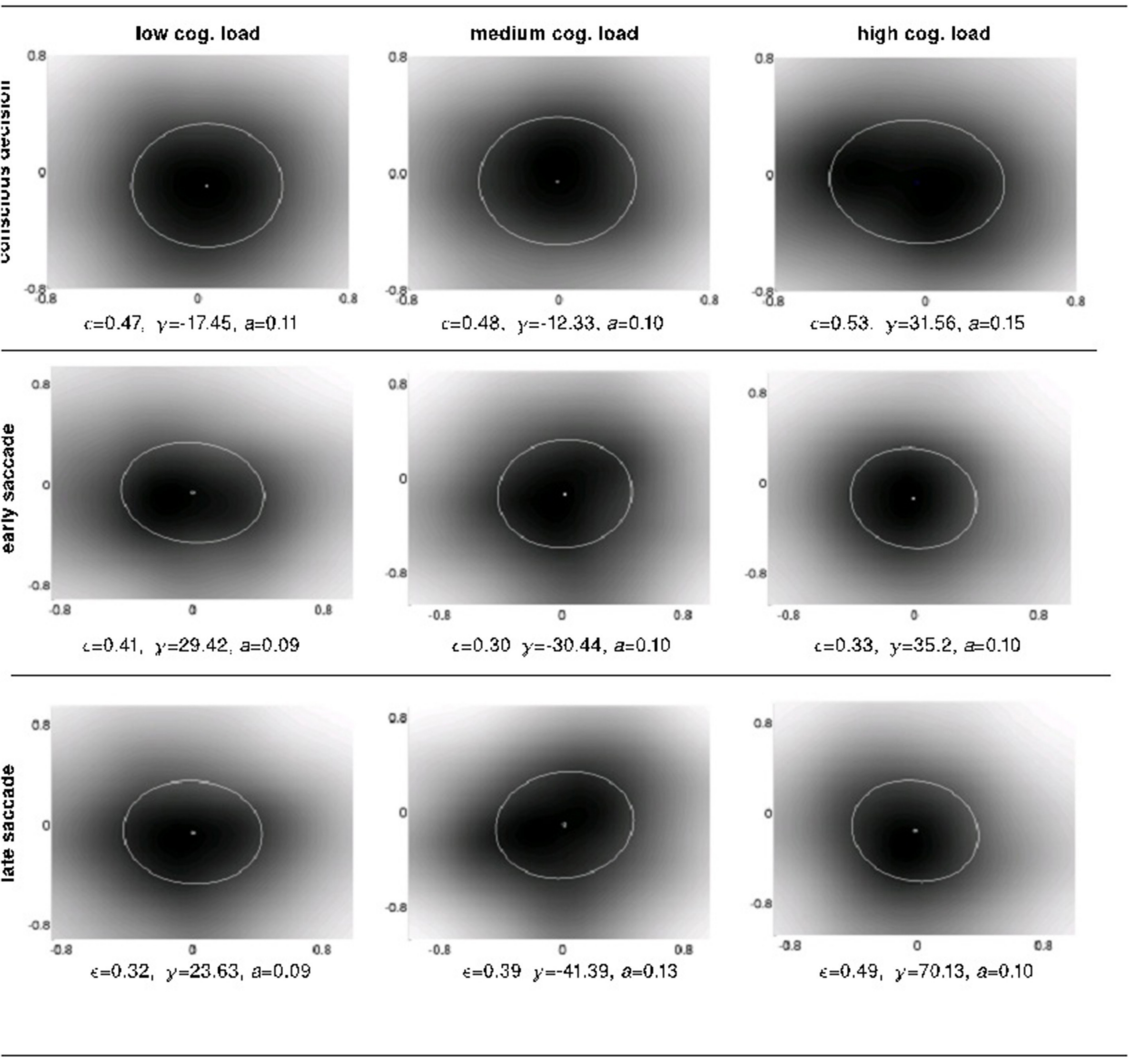
**The intra-subject psychophysical kernels as a function of response system and cognitive load**. We measured 4 properties of the ellipses fitted over the detection probability distributions (i.e., anticipatory fields): shift, eccentricity, orientation and area.

We designed a psychophysical reverse correlation analysis (Ahumada, 1996) to investigate the nature of the detected and the non-detected displacements. This technique provides a unique tool to uncover properties of internal representations and predictions (fast and slow saccades) and conscious decision strategies of individual participants in the perceptual task. The psychophysical kernel or anticipatory field (see Materials and Methods) represents the area (centered at the location of the item if there were no displacement) in which displacements of the item do not trigger responses, i.e., fast/slow saccades or button presses. Alternatively the anticipatory field could be thought of as the perceptive area where future data derived from the item are anticipated thus leading to no overt response to displacements. The intra-subject changes in the anticipatory fields shows a marked modulation by both the response system and the cognitive load condition considered (Figure 7).

To shed light onto the nature of the anticipations at work at different levels of perception, cognition and action, we investigated four different properties of the anticipatory fields computed separately for conscious detection, fast saccades and slow saccades: their area, eccentricity, shift and orientation. The area of the anticipatory field ellipse corresponds to the location of the non-detected displacements relative to the item's initial position. The orientation of the anticipatory field allows the definition of potential anticipatory biases in directions relative to the item's movement. If there were no bias in anticipations of future stimuli, the anticipatory fields should be circular (eccentricity 0) and centered at origin (shift 0), as a circular anticipatory field would signify a uniform distribution of the chances to detect displacements in all directions. The shift and eccentricity would similarly indicate a bias in the detection probability distribution. We normalized all displacements with respect to the item's movement direction, i.e., the positive x-axis (see Materials and Methods Figure 2). After the computation of the anticipatory fields for each subject and for each cognitive load, we analyzed intra-subject changes in the above properties of the anticipatory fields (Figure 7).

**Figure 8:**
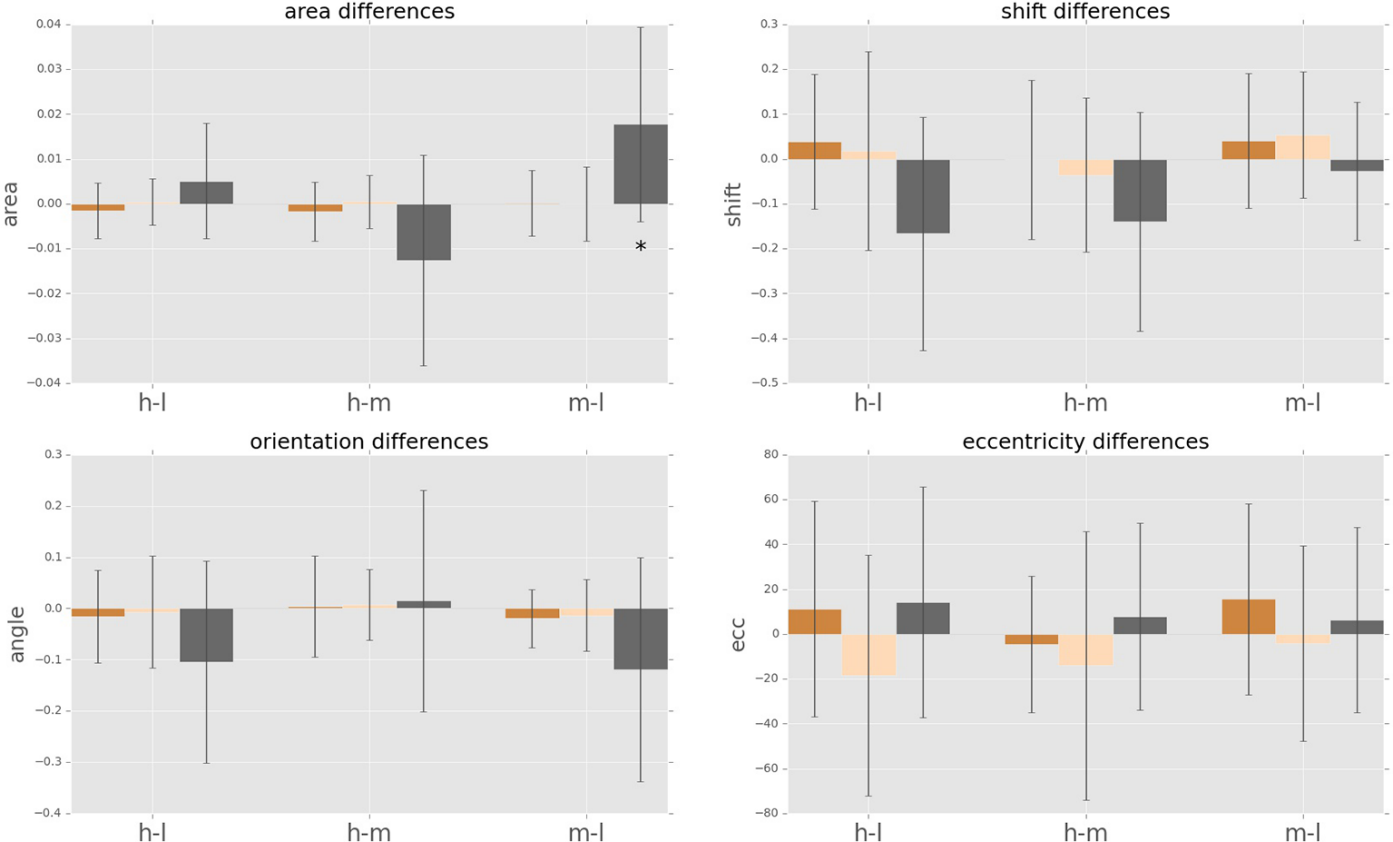
**Cognitive load dependent differences in intra-subject psychophysical kernels (i.e., anticipatory fields)**. Anticipatory field differences for the psychophysical kernels of fast saccades, slow saccades and conscious decision between the three cognitive load cases. “hl” indicates high load minus low load, “hm” indicates high load minus medium load, and “ml” indicates medium load minus low load. Star indicates significance of sign-test (*p* < 0.05). Differences in eccentricity, orientation and shift were not significant.

We observe a high variance in the inter-subject means for the above four parameters for all cognitive load conditions, suggesting distinct displacement detection baselines for each subject and making an intra-subject analysis more informative. For this, we computed the difference of the above four parameters for each subject between medium and low (m-l), high and medium (h-m) and high and low (h-l) cognitive loads. The anticipatory kernels were tested using a repeated measures MANOVA, as each subject completed three experimental conditions and we were testing multiple (four) parameters of the anticipatory kernel. A repeated measures MANOVA with a Greenhouse-Geisser correction determined that mean area of the anticipatory kernel representing conscious detections differed significantly between conditions [*F*_(1.460, 17.522)_ = 5.536, *p* = 0.02]. Post hoc tests using the Bonferroni correction revealed that an increase of cognitive load increased the kernel area, but only for the low to medium cognitive load condition (*p* = 0.028). For the medium to high condition the kernel area again started decreasing, however the trend was not significant (*p* = 0.273). These results seem to show a ceiling effect of the cognitive load on the kernel area increase. Medium cognitive load already caused maximum effects on anticipatory processes. Increasing the difficulty of the cognitive load task did not produce additional effects on anticipatory visual processes but only on the controlled voluntary processes (i.e., conscious detections). Therefore, we can observe no significant differences in kernel parameters between m-h conditions while still seeing a significant increase in reaction times for m-h [*F*_(1.455, 17.458)_ = 20.849, *p* = 0.009] and response accuracies [*F*_(1.455, 17.458)_ = 20.849, *p* = 0.009). We observed that there were no significant intra-subject changes in eccentricity, shift and orientation (Figure 8) in the conscious detections anticipatory kernel. These results were found only for conscious detections kernels, i.e., for the higher level processes, suggesting a top-down influence on movement anticipation. The fast and slow saccade kernels did not differ between conditions confirming their bottom-up nature (Fischer et al., 2007; Table 1).

**Table 1:** **Summary of behavioral results**.

	**Low cognitive load**	**Medium cognitive load**	**High cognitive load**
Response accuracy (Mean)	0.86 ± 0.11	0.80 ± 0.10	0.67 ± 0.22
Reaction time (Mean)	653 ms ± 127	654 ms ± 122	764 ms ± 134
Fast saccadic response (Median)	6.35	7.69	9.37
Slow saccadic response (Median)	17.19	13.3	15.0
Fast saccades followed (Median)	0.71	0.72	0.60
Slow saccades followed (Median)	1.0	0.76	0.63

Response accuracy and reaction times represent the conscious detection results (subject key-press) for the displacements. Fast and slow saccadic responses represent the percentage of displacements which were detected by the saccade. Saccadic detection is recorded if the gaze shifted towards the displacement location (shift vector cosine > 0.95). “Fast and slow saccades followed” represent the proportion (1.0 is a maximum) of the saccadic detections that were also followed by conscious detection (key-press).

## 5. Modeling

**Figure 9:**
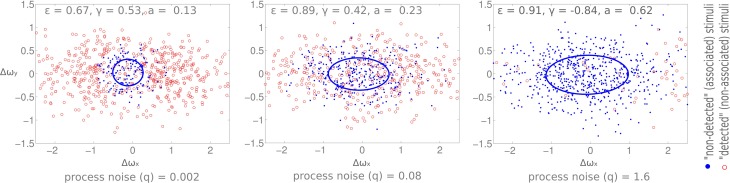
**Simulation results**. Eccentricity, orientation, and area of the anticipatory field are computed for varying process noise levels (q). The resultant shift was zero for all three cases. Process noise (q) is lowest on the leftmost plot and the highest on the rightmost one. X and Y axis are horizontal and vertical angular displacements (radians) respectively.

Our model investigates whether the observed changes in the anticipatory field are due to the cognitive load induced noise (see Materials and Methods section). Based on our earlier work on a self-contained model of bottom-up and top-down attention (Mathews et al., 2008, 2011), we use a probabilistic data association model from dynamic systems theory which uses a Kalman filter for the state update of individual items. A displacement that is not detected is modeled here as the association of sensory data to a known memory item. Analogously, a detected displacement is modeled as a sensory input not being associated to any items in memory. We model the cognitive load as process noise of the dynamic system which corresponds to the noise involved in the state updates of memory items (Bar-Shalom and Fortmann, 1988). Thereby we conjecture that an increase in cognitive load strains higher-level processes and this therefore induces higher noise in the state update of memory items. Indeed it has been shown that uncertainty in decision making correlates with an increase in the variability of firing in the prefrontal cortex (Marcos et al., 2013). We increased the process noise and observe the change in the anticipatory field properties (Figure 9). Following the empirical results, we observe an increase in the area (i.e., more missed displacements), and more significantly, an increase in the eccentricities when we compare the low load and high load conditions. Nevertheless, the change in process noise induces no change in shift and orientation as observed in our empirical data.

## 6. Discussion

We have investigated the question of how prediction affects different levels of perception and cognition from rapid saccades to conscious decisions. We have used manipulations of cognitive load to influence the coupling between these levels of processing. We observe that each level of processing we distinguished operates in specific constrained regions of sensory space defined as anticipatory fields confirming our theoretical prediction. Our results also suggest that cognitive load dynamically biases visual anticipation at the level of conscious decision making both in terms of the anticipatory field and the coupling with preceding more rapid attentional processing. We found a significant effect of cognitive load on the coupling between top-down and bottom-up processes, represented in the proportion of late saccadic detections followed by conscious detections (Figure 6). This proportion represents the amount of displacement events which were detected by a saccade divided by the amount of events which were detected by both the saccade and conscious response. The system controlling gaze behavior was responding to stimulus displacement with the same accuracy across conditions (no statistically significant differences in saccadic detection rates – Figure 6) while the amount of saccadic detections, which were also followed by conscious detections significantly decreased between conditions. This means that under low cognitive load condition the two systems were tightly coupled (median of saccadic detections followed by conscious detections = 1.0) while with increasing cognitive load the two systems became increasingly more uncoupled (median proportion in medium condition = 0.76 and in high condition = 0.63). Our finding that the violation of predictable stimulus movement (i.e., displacement) affected conscious and saccadic detections differently across conditions, suggests that predictions and their violations are not affecting all levels of processing equally, but rather influence the levels most relevant given the task demands. These results confirm that perceptual predictive hierarchies of the brain are not monolithic but rather potentially autonomous and encapsulated, being dynamically coupled or uncoupled dependent on task requirements. Our results suggest that this dynamic coupling serves the maintenance of a coherent conscious scene, in our case the cognitive load task. With respect to the question whether the conscious scene is dominated by predictions or prediction errors, our results suggest that conceptualized in terms of the anticipatory field the conscious scene not only is defined by predictions but also maintains their underlying representations at the expense of sensory data and its associated errors, i.e., with increasing cognitive load the region of input perceptual space covered by the anticipatory field increases leading to a greater tolerance for exceptions in the expected input. Hence, this suggests that the conscious scene aims at maintaining its world model and its derived predictions with respect to a specifically selected part of the input space that is, in the experiment reported here, in the center of gaze and closely matched to the specific properties of the sensory stimulus. In parallel, rapid subconscious processes assess alternative interpretations, i.e., displacements that are not processed at the level of conscious decisions can still be detected by the early visual system as measured using saccadic responses, and potentially gain access to consciousness when discrepancies are large enough. These subconscious processes are able to satisfy the task requirements as they were defined here, i.e., detect displacements. Conversely, errors, such as observations that fall outside of the anticipatory field, rather than impact the content of the conscious scene, trigger an allocation of attentional resources and in this way a reconstitution of the content of perceptual processing and the conscious scene.

### 6.1. Empirical results

In our analysis we combined a displacement detection task together with the detection of fast and slow saccades and psychophysical reverse correlation. Fast and slow saccades were first observed in monkeys using gap and overlap tasks, where a bimodal distribution of saccadic latencies to single targets was reported (Fischer and Rampsberger, 1984). Top-down influences on slow saccades have been reported previously, while this effect for fast saccades remains unclear. Our experimental paradigm deviates significantly from Multiple Object Tracking (MOT), where visually identical items move on non-linear Brownian motion tracks and the task is to keep track of a specific item (Pylyshyn and Storm, 1988). Our stimulus was designed to investigate if linear motion cues are used for anticipation of movements. Our results, i.e., the decrease of slow saccades followed by conscious detections, suggest that fast attentional processing can perform this task independent of top-down conscious representational systems. However, it is possible that details of the task have reduced this effect. Saccadic responses were in theory not necessary for detection of the displacement, which was still detectable while subject was maintaining a fixation at the center of the screen. From this position it was possible to cover the whole screen within the parafoveal field of view, where sensitivity for motion cues is maintained (McKee and Nakayama, 1984). Thus, the optimal strategy could have been to detect displacements peripherally, rendering frequent saccades unnecessary for successful completion of the task. By maintaining the perceptual task load over trials, and independently varying the cognitive load using the extra verbal task, we were able to investigate the role of cognitive load in biasing visual anticipations. The existence and the influence of higher level processes on visual anticipation have been shown previously via top-down contextual influences on property attributions to objects (Tremoulet and Feldman, 2006), top-down attentional influences on object location perception (Tse et al., 2011), attentional facilitation of motion perception based on past object movements (Watanabe, 1998) and implicit perceptual anticipation triggered by statistical learning (Turk-Browne et al., 2010). Our concept of anticipatory fields, characterizes and quantifies this influence of higher-level processes on anticipation at different levels of perception. Our approach measures conscious and unconscious decision making and provides a method to quantitatively assess the effects of prediction on perception at different levels of processing, posing an alternative to other psychophysical approaches like metacontrast masking (Lau and Passingham, 2006). The recurring topic of anticipation in conscious perception has found strong evidence in experimental comparisons showing the overlap between the default state network and the associative predictions network (Bar, 2009).

### 6.2. Model results

We proposed data association as the underlying mechanism to explain our finding and used the JPDA algorithm to model the variation in the anticipatory field properties with changing cognitive load. The process noise of the Kalman process used for state prediction and estimation of the moving items captures the influence of cognitive load on the anticipatory fields (Figure 9). Our model can be extended to non-linear movements (Bailey et al., 2006) and also to non-spatial domains (Gärdenfors, 2000). Our probabilistic model of perception is supported by earlier seminal research suggesting that what we see is a statistical consequence of past experience rather than a representation of the retinal stimulus itself (Purves et al., 2001; Verschure, 2003). An interesting parallel to our findings is the effect created by magicians, where most tricks rely on the fact that the human mind is vulnerable to deceptions as it works with anticipations about the world (Kuhn et al., 2008). The anticipatory field proposal could explain the psychological phenomenon of inattentional blindness (Simons and Chabris, 1999), as the former defines the perceptual area inside which changes in the sensory input mostly go unnoticed. To fully understand the process of anticipation and its influences on consciousness, perception and action our probabilistic model needs to be complemented with more detailed neural models and physiology (Lamme et al., 2000) or brain imaging (Turk-Browne et al., 2010) to investigate potential neural correlates of anticipatory fields. We predict that the dynamic dissociation we observe between the processes of conscious decisions and fast saccades due to cognitive load will be mirrored in the dynamic coupling and uncoupling of frontal and parietal regions of the visual cortex.

### Conflict of interest statement

The authors declare that the research was conducted in the absence of any commercial or financial relationships that could be construed as a potential conflict of interest.

## Footnotes

^1^further we omit the time-subscripts of state space variables for the sake of clarity

## References

[B1] Ahumada A. J. Jr. (1996). Perceptual classification images from Vernier acuity masked by noise. Perception 26, 1831–1840.

[B2] Bailey T. Upcroft B. Durrant-Whyte H. (2006). Validation gating for non-linear non-gaussian target tracking, in Information Fusion, 2006 9th International Conference on (Florence), 1–6.

[B3] Bar M. (2007). The proactive brain: using analogies and associations to generate predictions. Trends Cogn. Sci. 11, 280–289. 10.1016/j.tics.2007.05.005 17548232

[B4] Bar M. (2009). The proactive brain: memory for predictions. Philos. Trans. R. Soc. Lond. Ser. B Biol. Sci. 364, 1235–1243. 10.1098/rstb.2008.0310 19528004 PMC2666710

[B5] Bar-Shalom Y. Fortmann T. E. (1988). Tracking and Data Association. Boston, MA: Academic Press.

[B6] Barlow H. (1972). Single units and sensation: a neuron doctrine for perceptual psychology? Perception 1, 371–394. 10.1068/p010371 4377168

[B7] Barnes G. Barnes D. Chakraborti S. (2000). Ocular pursuit responses to repeated, single-cycle sinusoids reveal behavior compatible with predictive pursuit. J. Neurophysiol. 84, 2340–2355. 11067977 10.1152/jn.2000.84.5.2340

[B8] Barsalou L. W. (2008). Grounded cognition. Ann. Rev. Psychol. 59, 617–645. 10.1146/annurev.psych.59.103006.093639 17705682

[B9] Blakemore S. J. Frith C. D. Wolpert D. M. (1999). Spatio-temporal prediction modulates the perception of self-produced stimuli. J. Cogn. Neurosci. 11, 551–559. 10.1162/089892999563607 10511643

[B10] Boly M. Garrido M. I. Gosseries O. Bruno M.-A. Boveroux P. Schnakers C.. (2011). Preserved feedforward but impaired top-down processes in the vegetative state. Science 332, 858–862. 10.1126/science.1202043 21566197

[B11] Camos V. Barrouillet P. (2004). Adult counting is resource demanding. Br. J. Psychol. Lond. Engl. 95(Pt1), 19–30. 10.1348/000712604322779433 15005865

[B12] Cisek P. Kalaska J. F. (2010). Neural mechanisms for interacting with a world full of action choices. Ann. Rev. Neurosci. 33, 269–298. 10.1146/annurev.neuro.051508.135409 20345247

[B13] Clark A. (2013). Whatever next? Predictive brains, situated agents, and the future of cognitive science. Behav. Brain Sci. 36, 181–204. 10.1017/S0140525X12000477 23663408

[B14] Cox D. Meyers E. Sinha P. (2004). Contextually evoked object-specific responses in human visual cortex. Science 304, 115–117. 10.1126/science.1093110 15001712

[B15] Craik K. J. W. (1943). The Nature of Explanation. Cambridge, UK: Cambridge University Press.

[B16] Dean P. Porrill J. (2008). Adaptive-filter models of the cerebellum: computational analysis. Cerebellum 7, 567–571. 10.1007/s12311-008-0067-3 18972182

[B17] Duff A. Verschure P. F. M. J. (2010). Unifying perceptual and behavioral learning with a correlative subspace learning rule. Neurocomputing 10, 1818–1830 10.1016/j.neucom.2009.11.048

[B18] Edelman G. (2001). Consciousness: the remembered present. Ann. N.Y. Acad. Sci. 929, 111–122. 10.1111/j.1749-6632.2001.tb05711.x 11349421

[B19] Eggert T. Ladda J. Straube A. (2009). Inferring the future target trajectory from visual context: is visual background structure used for anticipatory smooth pursuit? Exp. Brain Res. 196, 205–215. 10.1007/s00221-009-1840-3 19466400

[B20] Ekstrom L. B. Roelfsema P. R. Arsenaullt J. T. Bonmassar G. Vanduffel W. (2008). Bottom-up dependent gating of frontal signals in early visual cortex. Science 321, 414–417. 10.1126/science.1153276 18635806 PMC3011100

[B21] Fischer B. Rampsberger E. (1984). Human express saccades: extremely short reation times to goal directed eye movements. Exp. Brain Res. 57, 191–195. 10.1007/BF00231145 6519226

[B22] Fischer A. Sananbenesi F. Wang X. Dobbin M. Tsai L. (2007). Recovery of learning and memory is associated with chromatin remodelling. Nature 447, 178–182. 10.1038/nature05772 17468743

[B23] Fries P. Reynolds J. H. Rorie A. E. Desimone R. (2001). Modulation of oscillatory neuronal synchronization by selective visual attention. Science 291, 1560–1563. 10.1126/science.1055465 11222864

[B24] Friston K. (2005). A theory of cortical responses. Philos. Trans. R. Soc. Lond. Ser. B Biol. Sci. 360, 815–836. 10.1098/rstb.2005.1622 15937014 PMC1569488

[B25] Friston K. (2010). The free-energy principle: a unified brain theory? Nat. Rev. Neurosci. 11, 127–138. 10.1038/nrn2787 20068583

[B26] Gärdenfors P. (2000). Conceptual Spaces: The Geometry of Thought. Cambridge, MA: MIT Press.

[B27] Gregory R. L. (1961). The brain as an engineering problem, in Current Problems in Animal Behaviour, eds Thorpe H. Zangwill O. L. (Cambridge: Cambridge University Press), 307–330.

[B28] Grossberg S. (1980). How does a brain build a cognitive code? Psychol. Rev. 87, 1–51. 10.1037/0033-295X.87.1.1 7375607

[B29] Grossberg S. (2009). Cortical and subcortical predictive dynamics and learning during perception, cognition, emotion and action. Philos. Trans. R. Soc. B Biol. Sci. 364, 1223–1234. 10.1098/rstb.2008.0307 19528003 PMC2666707

[B30] Helmholtz H. V. (1860). Handbuch der Physiologischen Optik, Vol. & Trans. JPC Southall. New York, NY: Dover.

[B31] Hesslow G. (2002). Conscious thought as simulation of behaviour and perception. Trends Cogn. Sci. 6, 242–247. 10.1016/S1364-6613(02)01913-7 12039605

[B32] Hohwy J. (2012). Attention and conscious perception in the hypothesis testing brain. Front. Psychol. 3:96. 10.3389/fpsyg.2012.00096 22485102 PMC3317264

[B33] Hohwy J. Roepstorff A. Friston K. (2008). Predictive coding explains binocular rivalry: an epistemological review. Cognition 108, 687–701. 10.1016/j.cognition.2008.05.010 18649876

[B34] Kalman R. E. (1960). A new approach to linear filtering and prediction problems. Trans. ASME J. Basic Eng. 82, 35–45 10.1115/1.3662552

[B35] Kawato M. (1999). Internal models for motor control and trajectory planning. Curr. Opin. Neurobiol. 9, 718–727. 10.1016/S0959-4388(99)00028-8 10607637

[B36] Kiesel A. Kunde W. Hoffmann J. (2007). Mechanisms of subliminal response priming. Adv. Cogn. Psychol. 3, 307. 10.2478/v10053-008-0032-1 20517516 PMC2864965

[B37] Kuhn G. Amlani A. A. Rensink R. A. (2008). Towards a science of magic. Trends Cogn. Sci. 9, 349–354. 10.1016/j.tics.2008.05.008 18693130

[B38] Lamme V. A. Supèr H. Landman R. Roelfsema P. R. Spekreijse H. (2000). The role of primary visual cortex (V1) in visual awareness. Vis. Res. 40, 1507–1521. 10.1016/S0042-6989(99)00243-6 10788655

[B39] Lau H. Rosenthal D. (2011). Empirical support for higher-order theories of conscious awareness. Trends Cogn. Sci. 15, 365–373. 10.1016/j.tics.2011.05.009 21737339

[B40] Lau H. C. Passingham R. E. (2006). Relative blindsight in normal observers and the neural correlate of visual consciousness. Proc. Natl. Acad. Sci. U.S.A. 103, 18763–18768. 10.1073/pnas.0607716103 17124173 PMC1693736

[B41] Laurens J. Meng H. Angelaki D. E. (2013). Computation of linear acceleration through an internal model in the macaque cerebellum. Nat. Neurosci. 16, 1701–1708. 10.1038/nn.3530 24077562 PMC3818145

[B42] Lisberger S. Morris E. Tychsen L. (1987). Visual motion processing and sensory-motor integration for smooth pursuit eye movements. Ann. Rev. Neurosci. 10, 97–1292. 10.1146/annurev.ne.10.030187.000525 3551767

[B43] Marcos E. Pani P. Brunamonti E. Deco G. Ferraina S. Verschure P.. (2013). Neural variability in premotor cortex is modulated by trial history and predicts behavioral performance. Neuron 78, 1–7. 10.1016/j.neuron.2013.02.006 23622062

[B44] Mathews Z. i Badia S. B Verschure P. F. (2008). Intelligent motor decision: from selective attention to a bayesian world model, in Paper Presented at the Intelligent Systems, is '08. 4th International IEEE Conference (varna).

[B45] Mathews Z. i Badia S. B. Verschure P. F. (2011). PASAR: an integrated model of prediction, anticipation, sensation, attention and response for artificial sensorimotor systems. Inf. Sci. 186, 1–19 10.1016/j.ins.2011.09.042

[B46] McKee S. P. Nakayama K. (1984). The detection of motion in the peripheral visual field. Vis. Res. 24, 25–32. 10.1016/0042-6989(84)90140-8 6695503

[B47] Merker B. (2005). The liabilities of mobility: a selection pressure for the transition to consciousness in animal evolution. Conscious. Cogn. 14, 89–114. 10.1016/S1053-8100(03)00002-3 15766892

[B48] Montague P. R. Dayan P. Person C. Sejnowski T. J. (1995). Bee foraging in uncertain environments using predictive Hebbian learning. Nature 377, 725–728. 10.1038/377725a0 7477260

[B49] Mudrik L. Breska A. Lamy D. Deouell L. Y. (2011). Integration without awareness: expanding the limits of unconscious processing. Psychol. Sci. 22, 764–770. 10.1177/0956797611408736 21555524

[B50] Nir Y. Tononi G. (2010). Dreaming and the brain: from phenomenology to neurophysiology. Trends Cogn. Sci. 14, 88–100. 10.1016/j.tics.2009.12.001 20079677 PMC2814941

[B51] Pally R. (2005). Non-conscious prediction and a role for consciousness in correcting prediction errors. Cortex 41, 643–662. 10.1016/S0010-9452(08)70282-X 16209328

[B52] Press W. H. Teukolsky S. A. Vetterling W. T. Flannery B. P. (1992). Numerical Recipes in C (2nd Edn.): the Art of Scientific Computing. New York, NY: Cambridge University Press.

[B53] Purves D. Lotto R. B. Williams S. M. Nundy S. Yang Z. (2001). Why we see things the way we do: evidence for a wholly empirical strategy of vision. Philos. Trans. R. Soc. Lond. Ser. B Biol. Sci. 1407, 285–297. 10.1098/rstb.2000.0772 11316481 PMC1088429

[B54] Pylyshyn Z. W. Storm R. W. (1988). Tracking multiple independent targets: evidence for a parallel tracking mechanism^*^. Spat. Vis. 3, 179–197. 10.1163/156856888X00122 3153671

[B55] Rao R. P. Ballard D. H. (1999). Predictive coding in the visual cortex: a functional interpretation of some extra-classical receptive-field effects. Nat. Neurosci. 2, 79–87. 10.1038/4580 10195184

[B56] Revonsuo A. (1995). Consciousness, dreams and virtual realities. Philos. Psychol. 8, 35–58 10.1080/09515089508573144

[B57] Revonsuo A. (2006). Inner Presence: Consciousness as a Biological Phenomenon. Cambridge, MA: MIT Press.

[B58] Simons D. J. Chabris C. F. (1999). Gorillas in our midst: sustained inattentional blindness for dynamic events. Perception 9, 1059–1074. 10.1068/p2952 10694957

[B59] Spratling M. W. (2010). Predictive coding as a model of response properties in cortical area v1. J. Neurosci. 30, 3531–3543. 10.1523/JNEUROSCI.4911-09.2010 20203213 PMC6634102

[B60] Summerfield C. Egner T. Greene M. Koechlin E. Mangels J. Hirsch J. (2006). Predictive codes for forthcoming perception in the frontal cortex. Science 314, 1311–1314. 10.1126/science.1132028 17124325

[B61] Tobii Technology (2014). Available online at: http://www.tobii.com/.

[B62] Tolman E. C. (1932). Purposive Behavior in Animals and Men. Oakland, CA: University of California Press.

[B63] Tremoulet P. D. Feldman J. (2006). The influence of spatial context and the role of intentionality in the interpretation of animacy from motion. Percept. Psychophys. 6, 1047–1058. 10.3758/BF03193364 17153197

[B64] Tse P. U. Whitney D. Anstis S. Cavanagh P. (2011). Voluntary attention modulates motion-induced mislocalization. J. Vis. 11:12. 10.1167/11.3.12 21415228 PMC3575214

[B65] Turk-Browne N. B. Scholl B. J. Johnson M. K. Chun M. M. (2010). Implicit perceptual anticipation triggered by statistical learning. J. Neurosci. 30, 11177–11187. 10.1523/JNEUROSCI.0858-10.2010 20720125 PMC2947492

[B66] Verschure P. (2003). A real-world rational agent: unifying old and new AI. Cogn. Sci. 27, 561–590 10.1207/s15516709cog2704/1

[B67] Verschure P. (2013). Formal minds and biological brains ii: from the mirage of intelligence to a science and engineering of consciousness. IEEE Expert 28, 33–36.

[B68] Verschure P. F. (2012). Distributed adaptive control: a theory of the mind, brain, body nexus. Biol. Insp. Cogn. Archit. 1, 55–72 10.1016/j.bica.2012.04.005

[B69] Verschure P. F. Kröse B. J. Pfeifer R. (1992). Distributed adaptive control: the self-organization of structured behavior. Robot. Auton. Syst. 9, 181–196 10.1016/0921-8890(92)90054-3

[B70] Verschure P. F. M. J. Pennartz C. M. A. Pezzulo G. (2014). The why, what, where, when and how of goal-directed choice: neuronal and computational principles. Philos. Trans. R. Soc. Lond. B Biol. Sci. 369:20130483. 10.1098/rstb.2013.0483 25267825 PMC4186236

[B71] Victor J. D. (2005). Analyzing receptive fields, classification images and functional images: challenges with opportunities for synergy. Nat. Neurosci. 8, 1651–1656. 10.1038/nn1607 16306893 PMC1622929

[B72] Watanabe T. M. I. (1998). Task-dependent influences of attention on the activation of human primary visual cortex. Proc. Natl. Acad. Sci. U.S.A. 19, 11489–11492. 10.1073/pnas.95.19.11489 9736764 PMC21670

[B73] Winges S. A. Soechting J. F. (2011). Spatial and temporal aspects of cognitive influences on smooth pursuit. Exp. Brain Res. 211, 27–36. 10.1007/s00221-011-2638-7 21442218 PMC3163841

[B74] Wyss R. König P. Verschure P. F. J. (2006). A model of the ventral visual system based on temporal stability and local memory. PLoS Biol. 4:e120. 10.1371/journal.pbio.0040120 16605306 PMC1436026

